# High Mortality among Premature Neonates with Positive Blood Culture Neonatal Sepsis in a Tertiary Hospital, Tanzania: A Call for Action

**DOI:** 10.3390/children8111037

**Published:** 2021-11-11

**Authors:** Delfina R. Msanga, Fatema Parpia, Eveline T. Konje, Adolfine Hokororo, Stephen E. Mshana

**Affiliations:** 1 Department of Pediatrics and Child Health, Weill Bugando School of Medicine, Catholic University of Health and Allied Sciences, Mwanza 1464, Tanzania; fparpia16@gmail.com (F.P.); adolfineh@gmail.com (A.H.); 2 Department of Public Health, Weill Bugando School of Medicine, Catholic University of Health and Allied Sciences, Mwanza 1464, Tanzania; ekonje28@yahoo.com; 3 Department of Microbiology and Immunology, Weill Bugando School of Medicine, Catholic University of Health and Allied Sciences, Mwanza 1464, Tanzania; stephen72mshana@gmail.com

**Keywords:** preterm, vital signs, neonatal sepsis, mortality

## Abstract

Well-documented vital signs are key in the prediction of sepsis in low- and middle-income countries. We determined prevalence, associated factors, and outcomes of positive blood culture sepsis in premature neonates at Bugando Medical Centre Mwanza, Tanzania. Temperature, oxygen saturation, heart rate, respiratory rate, and random blood glucose were repeatedly recorded at admission, 8 h, and 24 h in all 250 neonates enrolled. Clinical and microbiological data were collected from patient records followed by descriptive data analysis. The mean age of the neonates was 3 ± 5.2 days, with the majority (90%) aged <10 days. The prevalence of positive blood culture sepsis was 21.2% (95% CI: 16.1–26.2). The fluctuation of the random blood glucose (RBG) (aOR = 1.34, 95% CI: (1.07–1.67), *p* = 0.010), low oxygen saturation (aOR = 0.94, 95% CI: (0.88–0.99), *p* = 0.031), premature rupture of membrane aOR = 4.28, 95% CI: (1.71–10.71), *p* = 0.002), gestational age < 34 weeks (aOR = 2.73, 95% CI: (1.20–6.24), *p* = 0.017), and home delivery (aOR = 3.90, 95% CI: (1.07–14.19), *p* = 0.039) independently predicted positive blood culture. Significantly more deaths were recorded in neonates with a positive blood culture than those with a negative blood culture (32.1% vs. 5.1%, *p* < 0.001). In limited-resource settings, clinicians should use the vital signs and clinical information to initiate timely sepsis treatment among preterm neonates to prevent deaths and other morbidities.

## 1. Introduction

Globally, mortality in children below five years of age has decreased from 5 million in 1999 to 2.5 million in 2017. Despite this achievement, there is still a high mortality rate, with about 44% of under five deaths occurring within the neonatal period [1,2]. The majority (98%) of these deaths are reported in low- and middle-income countries (LMIC). In LMIC, sub-Saharan Africa has the highest risk of death, with the least progress in reducing the neonatal mortality rate [3]. In this region, the three leading causes of neonatal deaths are birth asphyxia, sepsis and premature birth account for 93% of deaths [3].

Sepsis is an important cause of morbidity and mortality in neonates and has been described as a clinical syndrome that is characterized by signs and symptoms of infection with or without accompanying bacteremia [4,5]. The risk of sepsis increases when the birth weight and gestational age are low due to low levels of circulating immunoglobulin G and an immature epithelial barrier and invasive procedural devices in premature neonates [6]. Clinical features alone are non-specific and are inefficient in diagnosing sepsis, especially in preterm neonates, and culture results take more than 48 h and/or may be unavailable in several health facilities in LMIC [4,7], making the management of neonatal sepsis in LMIC more challenging. In this situation, neonatal sepsis management is based on culture-independent algorithms of risk factor-based scoring systems and early warning signs [4,8]. Risk factors that have been used to select neonates for laboratory evaluation in LMIC include a lack of antenatal care, home deliveries, unhygienic and unsafe delivery practices, poor cord care, premature rupture of membrane (PROM), prematurity, and lethargy, to mention a few [9,10,11,12]. Early warning signs such as changes in temperature, heart rate, and respiratory rate have been used to identify neonates who are at high risk or who are experiencing deteriorating conditions [13] for prompt management. Early warning signs in combination with sepsis predictors are not well developed and utilized for neonatal care in many LMIC. Low temperature and desaturation are examples of indications of clinical deterioration, but their definition changes during admission and are neither analysed nor used in combination with other sepsis-related risk factors for the prediction of sepsis in preterm neonates. In addition, there is limited research exclusively involving premature neonates with regard to the utilization of changes in vital signs to appropriately manage neonatal sepsis in LMIC. In this study, we documented the prevalence, predictors, and outcomes of positive blood culture sepsis in premature neonates who had been admitted to neonatal units in a tertiary hospital in the city of Mwanza, Tanzania.

## 2. Materials and Methods

### 2.1. Study Design, Duration and Study Area

Neonatal data from between January and September 2020 were collected and analyzed. The data involved all preterm neonates who had been admitted to the neonatal ICU (NICU) and the neonatal ward in the pediatric department at Bugando Medical Center (BMC), Mwanza, Tanzania. BMC is a zonal consultant and Catholic University of Health and Allied Sciences teaching hospital with a bed capacity of 950. It caters the Lake Zone, which has a population of 16 million, comprising people from the Kagera, Geita, Mwanza, Shinyanga, Simiyu, and Mara regions. The neonatal unit is divided into the NICU and neonatal ward. The neonatal ward has 60 baby cots, with an average of 30 admissions in a week, and the neonatal ICU has 15 baby cots, with average admission of 5 neonates in a week.

### 2.2. Sample Size Estimation, Sampling Technique

The study aimed to enroll 183 neonates as the minimum sample size based on the Yamane Taro (1967) formula [14]. All of the preterm neonates who were admitted to the neonatal units between January and September 2020 were eligible for the study. This study included preterm neonates with clinical signs and symptoms of neonatal sepsis according to the WHO guidelines for sepsis in young infants [15] and who available data for the culture and sensitivity results. Medical records of neonates with unclear history, no vital signs records, and no completed culture were excluded. Medical records were retrieved serially until the desired sample size was reached.

### 2.3. Study Population and Data Collection

Neonatal medical records were extracted from all preterm neonates aged less than 28 days who had been admitted to the neonatal units of the BMC. Preterm neonates were defined as babies who were born alive at less than 37 completed gestation weeks and/or babies who had birth weight of less than 2500 gm [16]. A sepsis case was defined as a preterm neonate with clinical signs and symptoms of neonatal sepsis [17], with documented evidence of blood taken for culture. In these units, pediatric monitor machines were used to collect vital signs such as heart rate (H/R) and oxygen saturation (SPO2). Temperature and random blood glucose (RBG) were measured using a digital thermometer (OMRON Health Care, Tokyo, Japan) and a glucometer (One Touch, Milpitas, CA, United State), respectively. All of the vitals were documented on the neonates’ files. Using the WHO guidelines for sepsis in young infants [15], a data collection tool was designed and was used to retrieve socio-demographic and relevant clinical information from patient records that was related to neonatal sepsis. The information in the patient files was documented by residents who were involved in taking the history, the physical examination, and management of the neonates in these units and who were under the supervision of the pediatrician responsible for the unit.

### 2.4. Microbiological Analysis

Blood samples were processed following the BMC microbiology laboratory standard operating procedures (SOPs). Bacteria identification was carried out by conventional biochemical methods [18]. In case of uncertainty, the analytical profile index (API) 20E/20NE (bioMérieux, Marcy-l’Etoile, France) was used to confirm the identification [19]. The BMC clinical microbiology laboratory is accredited according to ISO 15189 by the Southern African Development Community Accreditation Service (SADCAS), an accreditation body with the registration number MD 002.

### 2.5. Data Management and Analysis

The medical files of the neonates were reviewed, and data were extracted using the data collection tool that had been customized in the Epicollect5 (https://five.epicollect.net/, accessed on 10 October 2020). Descriptive continuous data from the neonates such as birth weight (in grams), temperature (in °C), oxygen saturation (in %), random blood glucose (in mmol/L), respiratory (count), and pulse rate (count) were summarized using mean and standard deviation (sd), while Apgar scores were summarized using median and interquartile range (IQR). Categorical variables such as sex and survival status were reported as proportions. The differences in the characteristics and vital signs between neonates with positive and negative blood cultures were determined using student’s *t* test/Wilcoxon rank sum for continuous variables and the chi squared test for the categorical variables. Descriptive statistics were presented based on the blood culture results. The generalized estimating equation (GEE) was used to determine the significant predictors of sepsis based on the blood culture results using data from all time points (0 h, 8 h, 24 h). The GEE model was applied to account for the data dependence at different time points. The odds ratio was used to measure the associations and to report data with 95% confidence intervals. *p* values of 0.05 were considered to be statistically significant. All analyses were conducted using STATA version 15.

## 3. Results

### 3.1. Socio Demographic Characteristics of the Enrolled Preterm Neonates

During the study period, the data of 250 preterm neonates were retrieved from medical files and were analyzed. The mean age was 3 ± 5.2 days, with the majority (90%) of the neonates aged less than ten days. More than half of the neonates were male 143 (57.2%). A total of 12 (4.8%) of the neonates were delivered at home, and 41 (16.5%) required resuscitation after delivery (Table 1).

### 3.2. Preterm Neonates’ Characteristics Based on Blood Culture

The overall prevalence of sepsis based on a positive blood culture was 21.20% (95% CI; 16.54–26.75). There was an overall predominance of neonatal sepsis due to Gram-negative bacteria (72%) compared to sepsis due to Gram-positive bacteria (18%), *p* < 0.001. In Table 2, the significant differences seen for birth weight, Apgar score at one and five minutes, and survival status of the preterm neonates were observed by blood culture status. Neonates with a positive blood culture had a slightly lower birth weight than their counterparts. Similarly, the Apgar scores at one and five minutes were slightly lower in the neonates by blood culture results. Neonatal mortality was statistically higher in the neonates with a positive blood culture compared to those who had a negative blood culture.

**Figure 1:**
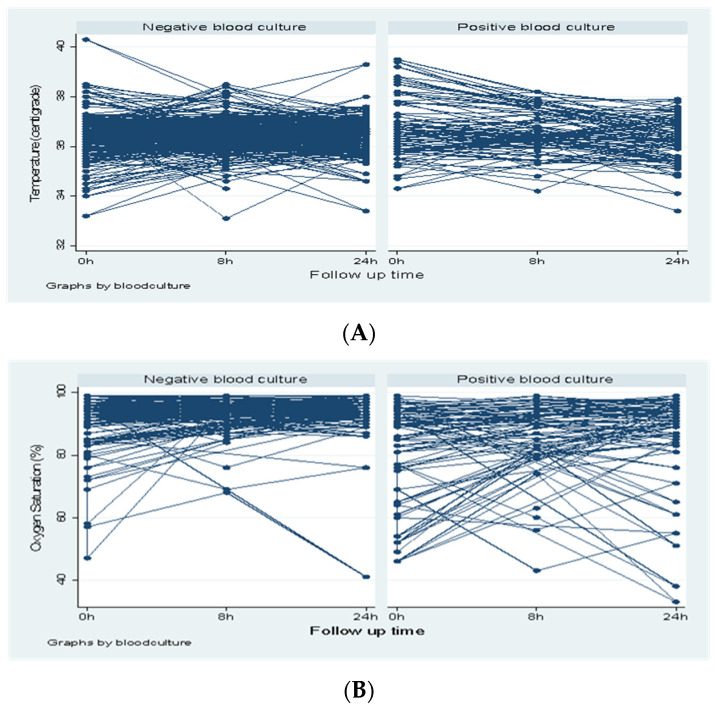

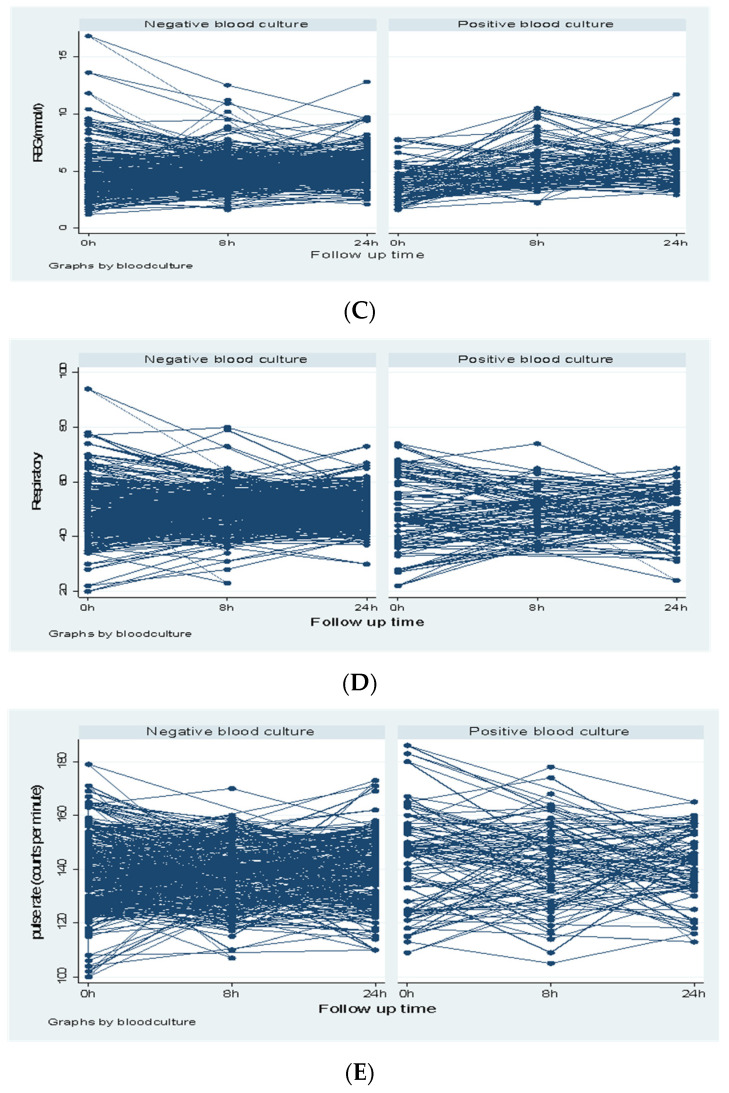
(**A**): Temperature records over 24 h based on blood sepsis; (**B**): oxygen saturation records over 24 h based on blood sepsis; (**C**): RBG records over 24 h based on blood sepsis; (**D**): respiratory rate records over 24 h based on blood sepsis; and (**E**): pulse rate records over 24 h based on blood sepsis.

The overall change in vital signs such, as temperature, oxygen saturation, random blood glucose, pulse rate, and respiratory at 0 h, 8 h, and 24 h, are presented in Figure 1A–E below. The overall trend in temperature over 24 hours was stable for the majority of the neonates with a negative blood culture, as shown on the dark area of Figure 1A. A significant mean temperature difference was observed at admission (36.43 °C vs. 36.79 °C, *p*-value = 0.01), with a slightly higher mean temperature observed among the neonates with a positive blood culture sepsis.

On average, neonates with no sepsis had stable oxygen saturation above 90% compared to neonates with confirmed sepsis, who showed oxygen saturation fluctuations over 24 h, as shown in Figure 1B. At every time point, the mean oxygen saturation was lower in neonates with confirmed sepsis, with significant differences being observed at 0 h (91.46% vs. 80.81%, *p*-value < 0.01), 8 h (91.86% vs. 83.91%, *p*-value < 0.01), and 24 h (92.66% vs. 82.08%, *p*-value < 0.01).

In Figure 1C, it was observed that the RBG remained stable over 24 h, with mean = 4.57 mmol/L at admission, 4.99 mmol/L at 8 h, and 5.11 mmol/L at 24 h for neonates with no sepsis compared to those with confirmed sepsis, who showed a mean RBG of 3.89 mmol/L, 6.37 mmol/L, and 5.61 mmol/L, respectively.

No difference was observed in respiratory count over the course of 24 h, as shown in Figure 1D. Lastly, the pulse rate differed significantly between neonates with and without sepsis. Those without confirmed sepsis had a stable trend of around 139 over 24 h, whereas neonates with sepsis were observed to have a significantly higher mean pulse rate of 146.21 vs. 139.49 at 0 h, but the mean pulse rate started dropping within 8 h to 143.19 and to 141.96 at 24 h, with no statistical difference between the groups shown in Figure 1E.

### 3.3. Maternal and Clinical Characteristics during Delivery

Of the neonates with confirmed sepsis, all most three quarters had mothers who had delivered at a gestational age earlier than 34 weeks compared to only half of those with negative results. The odds of having a positive blood culture were higher if the neonate had a lower gestational age. The odds of sepsis were four times higher among neonates born at a gestational age between 28–32 weeks, as per Table 3 below. Mothers who experienced PROM, fever, and who had delivered their newborns at home were more likely to be positive than their counterparts. Furthermore, assisted vaginal delivery exposed neonates to sepsis, as the odds of a positive culture were twice as high as they were for those who had undergone caesarian section.

### 3.4. Factors Associated with Confirmed Sepsis (Positive Blood Culture)

Model 1 looked at the maternal factors associated with blood culture, which is represented in Table 4. It was observed that a lower gestational age (28–32) in weeks, PROM, and home delivery were significantly associated with confirmed sepsis among preterm neonates. This indicates that the odds of a positive blood culture were higher among neonates born with a gestational age of 28–32 weeks compared to those born at 34–36 weeks. For those whose mothers experienced PROM or who were delivered at home, the odds of a positive blood culture were significantly higher than it was for their counterparts. In model 2, the odds of a positive blood culture were higher with increasing RBG, which was observed at 8 h, whereas a lower oxygen saturation at 0 h that remained lower over 8 h and 24 h was statistically associated with positive results among neonates. Vital signs such as temperature, respiratory, and pulse rate were not associated with confirmed sepsis at any time point in the multivariate analysis level.

### 3.5. Pathogens and Resistance Profiles

**Figure 2:**
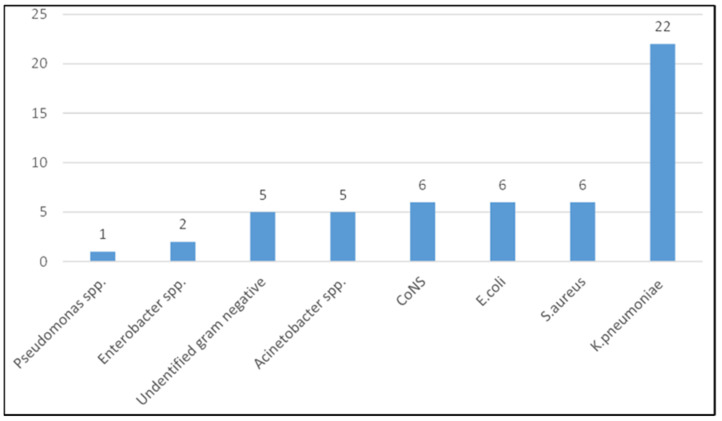
Pathogens isolated from 53 neonates with confirmed sepsis.

A total of 53 isolates were isolated; Gram-negative bacteria 41/53 (77.4%) formed the majority of the isolates (Figure 2). The most frequently isolated bacteria was *Klebsiella pneumoniae*, 22 (41.5%). For the Gram-negative bacteria (*n* = 41), resistance to antibiotics was tested (AMP: ampicillin, SXT: trimethoprim/sulfamethoxazole, AMC: amoxicillin/clavulanic acid, CN: gentamicin, CIP: ciprofloxacin, CRO: ceftriaxone, CTX: cefotaxime, TZP: piperacillin/tazobactam, MEM: meropenem, AK: amikacin) and ranged from 0.0% for gentamicin to 97.6% for ampicillin, Table 5). While the Gram-positive isolates (*Staphylococcus aureus* (6) and Coagulase negative Staphylococci (6)) were 100%, 83%, 66.7%, 58.0%, 50%, and 0.0% resistant to trimethoprim/sulfamethoxazole, erythromycin, ciprofloxacin, gentamicin, clindamycin, and vancomycin, respectively.

## 4. Discussion

Neonatal morbidity and mortality are key public health challenges in our local setting, with a huge percentage of deaths in the neonatal period being attributed to sepsis. This study aimed to assess the prevalence as well as the maternal and neonatal factors of preterm neonatal sepsis in order to contribute to tackling the burden of the illness and its related problems in LMIC. The reported prevalence in this study was found to be lower (21.20%) than previous studies in same setting [11,20] and other parts in East Africa [21] but higher than reports from developed countries [22,23]. The lower prevalence in the current study in this setting could be due to improvement of Infection Prevention and Control (IPC) and the fact that the data for the current study were extracted during the midst of the coronavirus disease 2019 (COVID-19) pandemic, where IPC was largely strengthened.

Most premature infants are treated empirically with antibiotics for either prolonged periods because they are at risk of early neonatal sepsis or are initiated on first dose of antibiotics in health facilities prior to urgent referral for further expertise management [24], putting them at an increased risk of MDR neonatal sepsis, as observed in the current study. Antibiotic drugs are administered for many reasons, including the relatively high incidence of EOS among preterm infants, with the high rate of mortality being attributable to infection and the frequency of clinical instability of premature neonates after birth [25]. The differences between high-income countries (HIC) and LMIC are due to the higher quality of life and strict IPC and antibiotic stewardship programmes in HIC compared to LMIC.

In the present study, preterm neonates with a positive blood culture had slightly a lower birth weight and lower Apgar scores at one and five minutes compared to their counterparts. These findings are consistent with various studies that have documented that the risk of neonatal sepsis increases with when the weight and gestational age are lower [26,27,28]. Preterm neonates have low levels of circulating immunoglobulin G and an immature epithelial barrier and are subject to invasive procedural devices; all of these have been documented as increasing the risk of neonatal sepsis [6]. The results of this study indicate that high mean temperature, hypoxemia, and fluctuation of RBG predicted a positive blood culture. In terms of the multivariate analysis, high RBG and hypoxemia remained significant as predictors of a positive blood culture. These vital signs have been documented as early warning signs before clinical deterioration [13,29]. The early warning signs that indicate that a patient is at a high risk of diseases or clinical deterioration are valuable tools for clinicians in guiding appropriate patient management [13] for the purposes of reducing associated morbidity and mortality. The combination of these important early warning signs and the clinical symptoms outlined in the WHO young infants study group [15] could be used areas where there are limited facilities to predict a positive blood culture and to initiate prompt empirical management that is supported by local susceptibility data.

This study found that maternal factors such as PROM, home delivery, and low a GA of less than 34 weeks were independently associated with culture-confirmed neonatal sepsis. We found that premature neonates who were born with a gestational age of less than 34 weeks had 2.73 odds of developing blood culture-proven neonatal sepsis as opposed to those born with a gestational age of more than 34 weeks. In a systematic review conducted in Ethiopia, it was reported that newborns with a low GA and a weight of less than 2.5 kg were 1.42 times more likely to develop neonatal sepsis infection compared to normal babies [30] and that death occurred more in those with low birth weight [30,31].

In the current study, mortality occurred in 27 (10.8%) of preterm neonates, and deaths were significant in neonates with positive blood culture results (*p* < 0.001). Mortality estimates varied depending on the gestational age of the infant; the lower the gestational age, the higher the risk of mortality due to the fact that premature neonates have an increased risk of infection and complications from being born too early compared to term neonates [32,33,34]. Delays in the diagnosis or treatment of sepsis may worsen clinical outcomes; hence, clinical care providers should have a high index of suspicion and should be knowledge of the proper treatment for possible sepsis.

In areas with limited resources, a combination of risks, early warning signs, and clinical symptoms could be used to predict a positive blood culture and to initiate prompt empirical management. The comprehensive prevention of neonatal sepsis requires a multi-interventional program that includes effec16tive maternal vaccination, the reduction of preterm delivery, and the usage of probiotics. Probiotics and lactoferrin have been investigated as potential preventative interventions in preterm neonates [35,36]. This warrants a future research area in this setting as a sustainable measure of prevention for adverse outcomes of neonatal sepsis in preterm neonates.

## 5. Limitation

The analysis was based on a review of medical records, so it was difficult to categorize early onset and late onset neonatal sepsis due to missing records. As such, we cannot rule out the possibility of our findings containing minimal bias.

## 6. Conclusions

Neonatal sepsis is an important cause of morbidity and mortality in this setting; PROM, low gestational age, and home delivery were found to predict a positive blood culture in premature neonates. In terms of physical findings, important vital signs such as desaturation during admission and mean changes in RBG significantly predicted positive blood culture results. We recommend the usage of these available tools for vital signs and proper clinical judgment for early the prediction of sepsis and for the initiation of proper empirical management to reduce adverse outcomes. Further larger studies are recommended in this group of neonates in order to analyze the contribution of MDR sepsis to the poor outcome of neonates.

## Figures and Tables

**Table 1:** Social demographic and characteristics of the admitted neonates.

Variable	Frequency/Mean	Percentage
Age	3 ± 5.2	
<10 Days	225	90.0
10–20 Days	16	6.4
21–28 Days	9	3.6
Sex		
Female	107	42.8
Male	143	57.2
Tribe		
Sukuma	93	37.2
Haya	25	10.0
Chagga	21	8.4
Others	111	44.4
Place Of Delivery		
Home	12	4.8
Hospital	238	95.2
Birth Weight		
Low birth weight	228	91.2
Very low birth weight	19	7.6
Extreme low birth weight	3	1.2
Gestational age (weeks)		
28–32	53	212
32–34	79	31.6
34–36	115	46
Less than 28	3	1.2
Resuscitation at birth		
No	207	83.5
Yes	41	16.5

**Table 2:** General characteristics of 250 neonates by blood culture results at tertiary hospital.

Factor	Positive Culture *n* (%)/Mean/Median (IQR)	Neg Culture *n* (%)/Mean/Median (IQR)	Total *n* (%)	OR/Mean (95% CI)	*p*-Value
Birth weight (g)	1777.02 ± 429.24	1930.97 ± 490.25		153.95 (8.24, 299.66)	0.04
Apgar score at 1 min	7 (7, 8)	8 (7, 8)		0.56 (0.42, 0.76)	<0.001
Apgar score at 5 min	9 (9, 10)	10 (9, 10)		0.64 (0.46, 0.90)	0.01
Sex					
Female	21 (39.62)	86 (43.65)	107 (42.80)	1	
Male	32 (60.38)	111 (56.35)	143 (57.20)	1.81 (0.64, 2.19)	0.59
Survival					
Dead	17 (32.08)	10 (5.08)	27 (10.80)	1	
Alive	36 (67.92)	187 (94.92)	223 (89.20)	0.11 (0.05, 0.27)	<0.001

**Table 3:** Maternal characteristics associated with neonatal positive blood culture.

Factor	Positive Culture *n* (%)	Negative Culture *n* (%)	Total *n* (%)	OR (95% CI)	*p*-Value
Gestation weeks					
34–36 weeks	14 (26.4)	101 (51.3)	115 (46.0)	1	
32–34 weeks	19 (35.9)	60 (30.5)	79 (31.6)	2.28 (1.07, 4.90)	0.034
28–32 weeks	20 (37.7)	36 (18.3)	56 (22.4)	4.01 (1.83, 8.77)	0.001
PROM					
No	40 (75.5)	184 (93.4)	224 (89.6)	1	
Yes	13 (24.5)	13 (6.6)	26 (10.4)	4.60 (1.98, 10.69)	<0.001
Maternal fever					
No	48 (20.2)	190 (79.8)	238 (95.2)	1	
Yes	5 (41.7)	7 (58.3)	12 (4.8)	2.83 (0.86, 9.32)	0.088
Place of delivery					
Hospital	46 (86.8)	192 (97.5)	238 (95.2)	1	
Home	7 (13.2)	5 (2.5)	12 (4.8)	5.84 (1.77, 19.29)	0.004
Meconium					
No	46 (86.8)	187 (94.9)	233 (93.2)	1	
Yes	7 (13.2)	10 (5.1)	12 (4.8)	2.85 (1.03, 7.89)	0.045
Mode of delivery					
C/S	19 (35.9)	114 (57.9)	133 (53.2)	1	
SVD	34 (64.2)	83 (42.1)	117 (46.8)	2.46 (1.31, 4.61)	0.005

**Table 4:** Factors associated with confirmed sepsis.

Model 1: Maternal Associated Factors				
		aOR	95% CI	*p* Value
Gestational age (weeks)	34–36 weeks	Ref		
	32–34 weeks	1.94	0.86, 4.36	0.109
	28–32 weeks	2.73	1.20, 6.24	0.017
PROM	No	Ref		
	Yes	4.28	1.71, 10.71	0.002
Place of delivery	Hospital	Ref		
	Home	3.90	1.07, 14.19	0.039
Meconium	No	Ref		
	Yes	1.56	0.45, 5.41	0.487
Mode of delivery	C/S	Ref		
	SVD	1.87	0.94, 3.74	0.076
**Model 2: Neonate Repeated Covariates Associated with Positive Blood Culture**				
Temperature	At admission	1.51	0.92, 2.47	0.102
RBG	At admission	0.81	0.65, 1.01	0.055
	At 8 h	1.34	1.07, 1.67	0.010
	At 24 h	1.21	0.92, 1.58	0.173
Oxygen saturation	At admission	0.94	0.88, 0.99	0.031
	At 8 h	0.99	0.90, 1.09	0.719
	At 24 h	0.96	0.85, 1.09	0.505

**Table 5:** Resistance profiles of Gram-negative bacteria.

Antibiotic	K. Pneumoniae (*n* = 22)	% Resistance	Other Gram Negative Bacteria (*n* = 19)	% Resistance	Total Gram Negative Bacteria (*n* = 41)	% Resistance
AMP	22	100.0	18	94.7	40	97.6
SXT	19	86.4	18	94.7	37	90.2
AMC	19	86.4	12	63.2	31	75.6
CN	21	95.5	8	42.1	29	70.7
CIP	13	59.1	9	47.4	22	53.7
CRO	19	86.4	16	84.2	35	85.4
CTX	19	86.4	16	84.2	35	85.4
TZP	12	54.5	9	47.4	21	51.2
MEM	0	0.0	3	15.8	3	7.3
AK	0	0.0	0	0.0	0	0.0

AMP: Ampicillin, STX: Trimethoprim-Sulphamethoxazole, AMC Amoxicillin-clavulunate, CN: Gentamicin, CP: Ciprofloxacin, CRO: Ceftriaxone, CTX: Cefotaxime, TZP: Piperacillin Tazobactam, MEM: Meropenem, AK: Amikacin.

## Data Availability

All data generated/analyzed during this study are included in this manuscript. Raw data can be obtained on request to the Director of Research and Publication, Catholic University of Health and Allied Sciences.

## Abbreviations

BPM	Breath Per Minute
BMC	Bugando Medical Centre
C/S	Caesarian Section
CUHAS	Catholic University of Health and Allied Sciences
EOS	Early Onset sepsis
GA	Gestational Age
IPC	Infection Prevention and Control
KCMC	Kilimanjaro Christian Medical University College
LOS	Late Onset sepsis
NICU	Neonatal Intensive Care Unit
PROM	Premature Rapture of Membrane
SVD	Spontaneous Vaginal Delivery
WHO	World Health Organization
